# Black tea bioactive phytoconstituents realign NRF2 for anticancer activity in lung adenocarcinoma

**DOI:** 10.3389/fphar.2023.1176819

**Published:** 2023-05-25

**Authors:** Suchisnigdha Datta, Anupam Bishayee, Dona Sinha

**Affiliations:** ^1^ Department of Receptor Biology and Tumor Metastasis, Chittaranjan National Cancer Institute, Kolkata, West Bengal, India; ^2^ College of Osteopathic Medicine, Lake Erie College of Osteopathic Medicine, Bradenton, FL, United States

**Keywords:** black tea, lung adenocarcinoma, chemoresistance, NRF2, KEAP1, redox signaling, treatment

## Abstract

Constitutive activation of nuclear factor erythroid 2-related factor 2 (NRF2) is pivotal in bestowing therapy resistance in cancer cells. Several phytochemicals have been reported with the potential of modulating NRF2. Therefore, it was hypothesized that NRF2-deregulated chemoresistance in lung adenocarcinoma (LUAD) may be counteracted by theaflavin-rich black tea (BT). A non-responsive LUAD cell line, A549, was the best sensitized towards cisplatin upon pre-treatment with BT. BT-mediated NRF2 reorientation was observed to be dependent on concentration and duration of treatment as well as on the mutational profile of NRF2 in A549 cells. Transient exposure of low-concentration BT hormetically downregulated NRF2, its downstream antioxidants, and drug transporter. BT also influenced the Kelch-like ECH-associated protein (KEAP1)-dependent cullin 3 (Cul3) and KEAP-1-independent signaling through epidermal growth factor receptor (EGFR) - rat sarcoma virus (RAS) - rapidly accelerated fibrosarcoma (RAF) - extracellular signal-regulated kinase 1/2 (ERK) - matrix metalloproteinase (MMP)-2 and MMP-9. The realignment of NRF2 in KEAP1-suppressed A549 cells enhanced the chemotherapeutic outcome. But a higher concentration of the same BT surprisingly upregulated NRF2 and its transcriptional targets with a subsequent decrease in the NRF2-regulatory machinery in NCI-H23 cells (a KEAP1-overexpressed LUAD cell line), ultimately resulting in a better anticancer response. The BT-mediated bidirectional NRF2 modulation was reconfirmed upon comparison with the action of a pharmacological NRF2 inhibitor, ML-385, in A549 and a known NRF2 activator, tertiary-butylhydroquinone, in NCI-H23 respectively. BT-mediated regulation of NRF2-KEAP1 and their upstream networks (EGFR/RAS/RAF/ERK) sufficed as a better anticancer agent than synthetic NRF2 modulators. Therefore, BT may be indicated as a potent multi-modal small molecule for increasing drug responsiveness in LUAD cells by maintaining NRF2/KEAP1 axis at an optimum level.

## 1 Introduction

Lung cancer continues to be the most lethal neoplasm of this millennium, and by 2035 it is expected to increase by 65.32% in about 40 countries across the globe (Luo et al., 2023). Non-small cell lung cancer (NSCLC) accounts for 85% of all diagnosed lung cancer cases. Lung adenocarcinoma (LUAD), the deadliest and most resistant form, constitutes 40% of NSCLCs (Chen et al., 2014). Aberrations in stress signaling pathways, such as nuclear factor erythroid 2-related factor 2 (NRF2)/Kelch-like ECH-associated protein (KEAP1), shape lung cancerization, especially adenocarcinoma, and subsequent prognosis (Zhang et al., 2019).

Under a healthy physiological state, NRF2 provides necessary protection to airway cells against pollutant-mediated constant oxidative stress, injury, and procarcinogenic alterations. Lung cancer cells may inherently evade the oxidative defense mechanisms with great efficiency (Liu et al., 2019). KEAP1, a redox-sensitive substrate adaptor for NRF2 is the key inhibitor of NRF2 which directly controls its cytosolic localization and nuclear transactivation. KEAP1-independent regulation of NRF2 includes epidermal growth factor receptor (EGFR)/rat sarcoma virus (RAS)/rapidly accelerated fibrosarcoma (RAF)/extracellular signal-regulated kinase 1/2 (ERK) signaling which activates NRF2 (Huo et al., 2014) and are also the driver genes of LUAD (Krall et al., 2017). NRF2 is directly involved in chemo-response because its transcriptional targets are responsible for the invasive gelatinolytic activity, drug-metabolizing, and transporting ability of the cancer cells (Shi et al., 2017; Matzinger et al., 2018). Mutations in these driver genes along with somatic mutations in NRF2/KEAP1, loss of heterozygosity, and other genetic/epigenetic alterations in this redox-regulatory system, are associated with aggressive progression and therapeutic failure in greater than 40% of LUAD cases (Barrera-Rodríguez, 2018).

Cisplatin, one of the standard platinum-based chemotherapeutic drugs causes electrophilic damage to neoplastic cells and is widely used for LUAD treatment. Chemoresistance to cisplatin may be explained by the hyperactivation of NRF2-targeted drug transporters and drug-detoxifying enzymes, especially in KEAP1-mutated LUAD cells (Silva et al., 2019).

Tea, prepared from the plant *Camellia sinensis,* is one of the most ancient yet popular health beverages worldwide. Tea phytochemicals are not only promising anticancer agents but also known NRF2 modulators (Datta et al., 2022). The predominant health benefits of tea lie in its canonical antioxidative behavior owing to its efficient scavenging ability of the reactive oxygen species (ROS). The major antioxidant polyphenols of black tea, namely, thearubigin (TR) and especially theaflavin (TF), show maximum anticancer potential because of their abundant hydroxyl groups (-OH) and excellent bioactivity (National Cancer Institute, 2010; Aggarwal et al., 2022). However, tea phytochemicals exhibit dual characteristics of anti-/pro-oxidative activity depending on the concentration/duration of exposure as well as the genetic profile and redox status of the malignant cells (Butt et al., 2014). Interestingly black tea-influenced toxicity specific to cancer cells is majorly oxidative damage (Koňariková et al., 2015). This dual redox regulation makes tea an ideal candidate for the modulation of the redox regulator NRF2. Moreover, TF can overcome cisplatin resistance when used as an adjuvant in ovarian cancer cells (Pan et al., 2018). Therefore, in this work, black tea (BT) phytochemicals by their selective ROS-dependent anticancer efficacy were explored for the maintenance of NRF2-mediated redox homeostasis in different KEAP1-mutated LUAD cell lines. The chemo-sensitizing efficiency of BT as an adjuvant was also tested in unresponsive LUAD cells.

## 2 Materials and methods

### 2.1 Chemicals

Roswell Park Memorial Institute (RPMI) 1,640 culture media, fetal bovine serum (FBS), antibiotics (penicillin 10,000 units/ml and streptomycin 10 mg/mL), and trypsin (0.25%) were procured from Invitrogen, Life Technologies Corporation (Grand Island, NY, United States). 3-(4,5-dimethylthiazol-2-yl)-2-5-diphenyl- 2H-tetrazolium bromide (MTT) was purchased from Amresco (Solon, OH, United States). 5,5,6,6′-tetrachloro-1,1′,3,3′tetraethyl benzimi-dazoyl carbocyanine iodide (JC-1) dye was purchased from Thermo Fisher Scientific (Insert City, MA, United States). Calcein-AM/PI double staining kit was purchased from Cayman (Ann Arbor, MI, United States). Cisplatin, etoposide, and BT (>80% theaflavin) were procured from Sigma-Aldrich (St. Louis, MO, United States). Gelatin sepharose was purchased from GE Healthcare (Uppsala, Sweden). NRF2 was procured from Cell Signaling Technology (Denver, MA, United States). KEAP1 and superoxide dismutase-1 (SOD-1) antibodies and protein ladder (10-245KD) were purchased from Abcam (Cambridge, United Kingdom). EGFR, p-EGFR, RAS, RAF, ERK, p-ERK, matrix metalloproteinase (MMP)-2, MMP-9, primary antibodies, fluorescein isothiocyanate (FITC)/phycoerythrin (PE)-tagged, and horse-radish peroxidase (HRP)-tagged secondary antibodies were purchased from Santa Cruz Biotechnology (Dallas, TX, United States). NAD(P)H: quinone oxidoreductase-1 (NQO-1), and multidrug resistance protein-1 (MRP-1) antibodies were procured from Novus Biologicals (Littleton, CO, United States). Polymerase chain reaction (PCR) kit, GoTaq qPCR Master Mix and reverse transcriptase (M-MLVRT) were purchased from Promega (Madison, WI, United States).

### 2.2 Cell culture and treatment

Cell lines, A549 and NCI-H23, were obtained with short tandem repeat DNA profile authentication from National Centre for Cell Sciences (Pune, India). BEAS-2B a normal bronchial epithelial cell line was a generous gift from Dr. Mainak Sengupta, University of Calcutta (Kolkata, India). A549 cells were cultured in RPMI supplemented with heat-inactivated FBS (10%), penicillin (100 U/mL), and streptomycin (100 mg/mL) at 37°C in a humidified atmosphere of 5% CO_2_ incubator. For NCI-H23, RPMI with low bicarbonate (1.5 M) supplements and no antibiotics was used keeping other conditions the same. BEAS-2B was routinely cultured in an LHC-9 medium.

Concentration and time-dependent studies of the cell lines were performed with BT for a wide concentration range (0.046–46 μg/ml)]and time range (3–72 h). Chemotherapeutic drugs cisplatin or etoposide were added (0–100 μM) individually or in combination with the most potent cytotoxic concentration of BT (0.46 μg/ml) for a varied period (24, 48, or 72 h) to study cytotoxicity.

Three different modes of combination treatment were followed as described below.

i) Concurrent-treatment: Cisplatin (1 μM) was either used in combination with etoposide (1 μM) or BT (0.46 μg/mL) (either cisplatin + etoposide or cisplatin + BT) and the treatment duration was 48 h.
ii) Pre-treatment/sensitization: Initial treatment with BT (0.46 μg/mL/6 h) was followed by the addition of cisplatin (1 μM) and the total treatment duration was 24 or 48 h.
iii) Post-treatment or potentiation: Initial cisplatin (1 μM) treatment was followed by the addition of BT (0.46 μg/mL/6 h) and the total treatment duration was 24 or 48 h

### 2.3 MTT assay

Exponentially growing cells (1×10^4^) were seeded in 96-well microplates for the MTT assay. After 24 h of growth, the cells were exposed to serial concentrations of the compounds for varied treatment periods. A separate lane was also kept for a time-matched control (A549/NCI-H23/BEAS-2B cells, without any treatment) and vehicle control (0.1% DMSO in case of BT and complete media for cisplatin or etoposide). The MTT solution was added to each well (1.2 mg/ml) and was kept in the dark for 4 h. The absorbance of the MTT-formazan product dissolved in DMSO was measured at 570 nm in a plate reader (Infinite M200, TECAN, Mannedorf, Switzerland). The calculation of cell viability (%) was done by considering the O.D. of control as 100%. All further experiments were performed with the cytotoxically most potent concentrations in NCI-H23 and A549, respectively.

### 2.4 JC-1 staining

The mitochondrial membrane-permeable fluorescent dye, JC-1, was used to monitor cellular health to study apoptosis. Positively charged red monomeric JC-1 (emission: 590 nm) can easily get accumulated as green polymeric JC-1 aggregate (emission: 529 nm) inside the electronegative environment of healthy mitochondria. A mitochondrial depolarization causes a red-to-green shift which results in a decrease in the red: green ratio. Treated and control JC-1-stained slides were visualized under a fluorescence microscope (Olympus BX53F, Tokyo, Japan) with red and green spectra using the software CellSens Standard 1.15 (Olympus, Tokyo, Japan).

### 2.5 Comet assay

To analyze the amount of DNA damage (double and single strand breaks) comet assay or alkaline single-cell gel electrophoresis was done in A549 cells (Datta and Sinha, 2019). Treated and control cells (1×10^4^) were layered on normal melting agarose-coated frosted slides, then lysed, and finally electrophoresed for 20 min (300 mA, 20 V). Slides were then soaked in neutralizing buffer, then stained with ethidium bromide (final concentration 40 μg/ml), and visualized under a fluorescence microscope (excitation: 518 nm and emission: 602 nm) (DM 4000B; Leica, Wetzlar, Germany). Tail DNA% (TD%) and olive tail moment (OTM) were calculated using the software Komet 5.5.

### 2.6 MDR assay

The modulation of cellular MDR machinery was studied in A549 cells using a non-radioactive multidrug resistance-specific Calcein-AM assay kit following the manufacturer’s protocol (Datta and Sinha, 2019). Glycerol-mount fluorescent-labeled cells were visualized under a fluorescence microscope (Olympus BX53F, Tokyo, Japan) with Calcein (excitation: 490 nm and emission: 520 nm) and PI (excitation: 535 nm and emission: 620 nm) using software CellSens Standard 1.15 (Olympus, Tokyo, Japan).

### 2.7 Live cell microscopy

BT-pre-sensitized cisplatin-treated cells and only cisplatin-treated cells were visualized for their dynamic changes in morphology and MRP1-expression. Cells were incubated with anti-MRP1 antibody followed by FITC-tagged anti-mouse secondary antibody in a CO_2_ incubator and washed with media before imaging. The real-time images were captured using live-cell microscopy (CytoSMART, Lux3 FL, Eindhoven, Netherlands) from 0 to 3 h of exposure with an interval of 1 min. The confluency and fluorescence intensity was measured using the in-built software (CytoSMART, Eindhoven, Netherlands) (Cytosmart CytoSMART, 2021).

### 2.8 Nuclear and cytosolic protein isolation

The separation of cytosolic and nuclear extracts was done according to a previously published protocol (Datta and Sinha, 2019). Cells were lysed using a hypotonic buffer. The nuclei were pelleted at 13,000 x g for 15 min, and subsequently, the cytoplasmic supernatant was aspirated. From the pellet, the nuclear fraction was extracted using nuclear lysis buffer along with nuclear storage buffer.

### 2.9 Western blotting

The cells were lysed using 1x radioimmunoprecipitation assay (RIPA) buffer containing protease inhibitors. Protein content was estimated by Lowry’s method (Lowry et al., 1951). Protein (50 µg) was separated on SDS-polyacrylamide (acrylamide-bis-acrylamide) gel and electroblotting was performed onto a polyvinylidene difluoride (PVDF) membrane. Membranes were incubated with primary antibodies (NRF2/KEAP1/EGFR/p-EGFR/RAS/RAF/ERK/p-ERK/NQO1/SOD1/MRP1) (1:2000 dilution) overnight at 4°C, washed thrice with tris buffer saline-tween 20 (TBS-T) and incubated with AP coupled anti-mouse or anti-rabbit or anti-goat secondary antibody for 90 min at 37°C (1:10,000). Bands were visualized using a colorimetric method in the Molecular Imager Gel-doc XR + Chemidoc UV system (Bio-Rad, Hercules, CA, United States) and were quantitated using ImageJ Launcher (version 1.4.3.67).

### 2.10 Immunocytochemistry

Cells were grown on coverslips placed into culture dishes and treated with BT, fixed in 100% cold methanol, and permeabilized with 0.1% Triton-X-100. After that, the coverslips were blocked in 4% bovine serum albumin (BSA) and were incubated overnight at 4°C with anti-NRF2, or anti-KEAP1 antibody (2.5 μg/ml) in PBS containing 1.5% BSA. This was followed by TBS washing, secondary antibody incubation, and further washing. The cells were finally stained with 3,3′-diaminobenzidine (DAB) for another 45 min in the dark, washed, mounted in DPX, and examined under a bright-field microscope (Olympus BX53F, Tokyo, Japan).

### 2.11 Co-immunolocalization

The functionality of many proteins depends on their subcellular localization and interaction with other proteins (e.g., NRF2 and KEAP1). To study the physically interacting proteins NRF2 and KEAP1 their double immunofluorescence staining was performed. The NCI-H23 and A549 cells (1.0×10^5^) were grown on coverslips in 35-mm plates and exposed to BT (2.3 μg/ml/12 h) or BT (0.46 μg/mL/6 h) respectively. The control and treated cells were fixed, permeabilized, and blocked same as immunocytochemistry till simultaneous incubation with the rabbit monoclonal anti-NRF2 primary antibody (1:400) and mouse monoclonal anti-KEAP1 primary antibody (1:1,000) at 4°C in a humidified chamber. After washing the samples were incubated with FITC-tagged mouse anti-rabbit secondary antibody and PE-tagged goat anti-mouse secondary antibody (1:2,500) and 0.2 μg/ml DAPI was used for nuclear counter-staining. For the co-localization assay, the glycerol-mounted slides were sequentially visualized under a fluorescence microscope (DM 4000B; Leica, Wetzlar, Germany) with FITC (excitation: 495 nm and emission: 519 nm), PE (excitation: 566 nm and emission: 574 nm) and DAPI (excitation: 359 nm and emission: 457 nm) channels.

Co-localization image analysis was done in the Fiji setting of ImageJ software (NIH, Bethesda, MD, United States) using the JACoP plugin (Bolte and Cordelières, 2006). Immunofluorescence intensities of NRF2 (FITC) and KEAP1 (PE) were analyzed for the two co-localization parameters, Pearson’s correlation coefficient (PCC) (Pearson, 1896), and Mander’s overlap coefficient (M1 and M2) (Manders et al., 1993). PCC determines the deviation of intensity of each pixel for a particular fluorophore against the other, M1 signifies the intensity of fluorophore A (FITC) overlapping with the above threshold intensity of fluorophore B (PE) and the opposite is for M2. The pixel distribution of each slide was displayed in a 2D scatter plot (PE on the *X*-axis and FITC on the *Y*-axis) obtained from the coloc-2 tool (Shrestha, 2019). Values were calculated from a minimum of 8 representative images per slide from where the region of interest (ROI) and the signal threshold was predetermined. Though values above 0 implied co-localization, a baseline of less than 0.5 represented a strong physical synapse.

### 2.12 Estimation of enzymatic activity of NRF2-transcriptional targets

NQO1 activity was investigated according to our previously standardized method where a decrease in NADH absorbance due to the presence of menadione was measured at 340 nm (Datta and Sinha, 2019) (Cu-Zn) SOD activity was determined at 420 nm by the ability of the enzyme to inhibit the autoxidation of pyrogallol in the presence of EDTA following our earlier publication (Datta and Sinha, 2019).

### 2.13 Gelatin zymography

The study of the gelatinolytic activity of MMP-2 and MMP-9 by in-gel zymography was done using the previously standardized method (Datta and Sinha, 2022). Secreted MMPs were concentrated from serum-free conditioned medium (SFCM) by binding to gelatin sepharose 4B beads and were subjected to zymography on 10% SDS-PAGE co-polymerized with 0.1% gelatin. 0.5% Coomassie Blue stain-free bands were visualized in the Molecular Imager Gel-doc XR + Chemidoc UV system (Bio-Rad, Hercules, CA, United States) and were quantitated using ImageJ Launcher (version 1.4.3.67).

### 2.14 Spheroid culture

To better understand solid tumor biology and the antitumor drug activity, a multi-cellular three-dimensional cell aggregate was formed. Cells A549/NCI-H23 were seeded in a 96-well ultra-low attachment plate with an initial cell number of 2,500 cells/well and maintained for 72 h. After 24 h, the A549 and NCI-H23 spheroids were treated with NRF2-inhibitor (ML-385) and NRF2-activator (t-BHQ) respectively. Spheroids were visualized under a bright-field inverted microscope (DM 4000B; Leica, Wetzlar, Germany). Spherical properties such as sphericity, compactness, and area: volume ratio were measured using the AnaSp software from the size-adjusted gray-scale images (Piccinini, 2015).

### 2.15 Semi-quantitative PCR and real-time PCR

Isolation of total RNA from cell lysate was performed using the trizol-chloroform extraction method. Conversion of total RNA to cDNA was done using oligo dT, M-MLV RT, and 5x buffer. The cDNA obtained was then amplified by polymerase chain reaction for 35 cycles with an initial hot start followed by denaturation, annealing, and extension at (94°C for 30°s; 55°C for 30°s; and 72°C for 90 s) using primers for human NFE2L2 [forward (ACA​CGG​TCC​ACA​GCT​CAT​C), reverse (TGT​CAA​TCA​AAT​CCA​TGT​CCT​G), Gene bank accession no. NM_001313903.2], human KEAP1 [forward (TTC​GCC​TAC​ACG​GCC​TC), reverse (GAA​GTT​GGC​GAT​GCC​GAT​G) Gene bank accession no. NM_012289.4]. Housekeeping genes, human *β*-actin [forward (GTC​CAC​CTT​CCA​GCA​GAT​GTG), reverse (GCA​TTT​GCG​GTG​GAC​GAT), Gene bank accession no. NM_001101.5], glyceraldehyde 3-phosphate dehydrogenase (GAPDH) [forward (GAA​GAC​GGG​CGG​AGA​GAA​AC), reverse (CCA​TGG​TGT​CTG​AGC​GAT​GT), Gene bank accession no. NM_001289745.3] were used for normalization. PCR products were analyzed in 2% agarose gel electrophoresis and finally visualized under the gel documentation system Molecular Imager Gel-doc XR + Chemidoc UV system (Bio-Rad, Hercules, CA, United States).

The real-time quantitation of RNA expression was done by GoTaq qPCR Master Mix. The cDNAs were amplified with the previously mentioned same specific primers for NRF2, KEAP1, and GAPDH (Light cycler 96, Roche). The relative level of gene expression was determined by the comparative threshold cycle (Ct) method based on the average Ct value of the gene of interest and the internal control from the triplicate set of samples (ΔCt). Next, the ratio of ΔCt treated vs. untreated samples was deduced (ΔΔCt). A log2^(-ΔΔCt) value expressed as the fold change was graphically plotted for each of the target genes.

### 2.16 Statistical analysis

The statistical significance between treated and control groups was determined using the one-way Analysis of Variance (ANOVA) followed by the Dunnett *t*-test, where the *p*-value was set at 0.05 to check the statistical difference between groups. Dunnett’s *t*-test treats one group as a control and treats all other groups against it. All the significances were calculated in comparison to the control and the *p*-value was marked accordingly if not mentioned otherwise. All results were computed and analyzed using the SPSS statistical software package 20.0 (SPSS, Chicago, IL, United States).

## 3 Results

### 3.1 Differential cytotoxicity of BT in different lung cell lines

**FIGURE 1:**
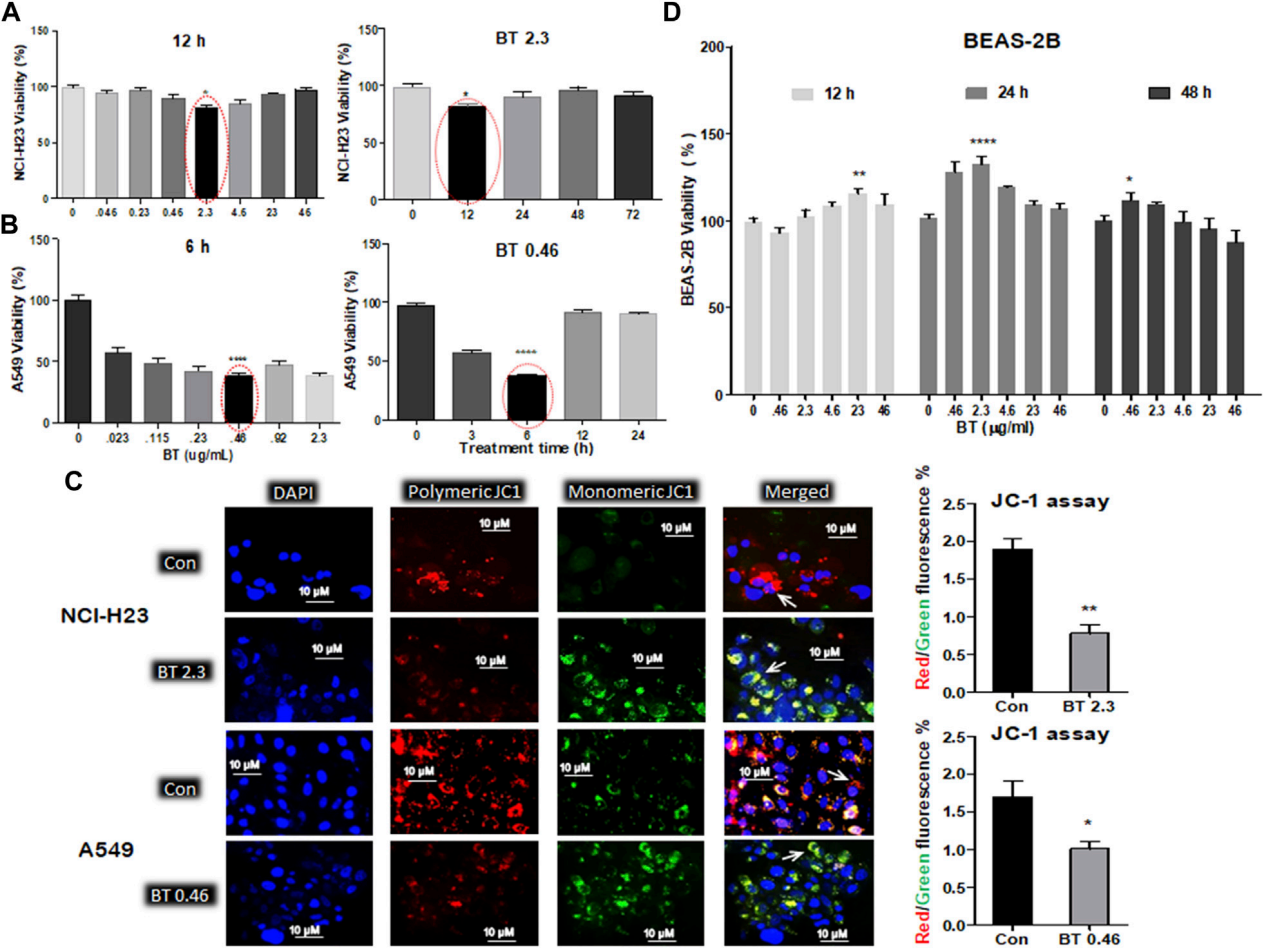
Differential cytotoxicity of BT in different lung cell lines. Concentration- and time-dependent effect of black tea extract (BT) on the viability of two lung cancer cell lines- NCI-H23 **(A)** and A549 **(B)**; effect of selected concentration of BT on the mitochondrial polarization of NCI-H23 and A549 cells **(C)** by JC-1 staining; concentration- and time-dependent effect of BT on the viability of normal lung cell line- BEAS-2B **(D)**. *p < 0.05, **p < 0.01, ***p < 0.001, and ****p < 0.0001, compared with the control if not mentioned otherwise. Abbreviations: BT 0.46, BT 0.46 μg/mL/6 h; BT 2.3, BT 2.3 μg/mL/12 h.

The various concentration/time ranges of BT were tested for their differential cytotoxicity in A549, NCI-H23, and BEAS-2B lung cell lines. Cytotoxicity was represented by a decrease in viability. In NCI-H23 highest cytotoxicity of 28% was observed with BT (2.3 μg/mL/12 h; Figure 1A), therefore, this was considered the most potent cytotoxic concentration in NCI-H23. However, a “U”-shaped pattern of cytotoxicity was seen with BT (0.046–46 μg/mL) concentration range in NCI-H23 cells.

In A549, a very low and transient concentration of BT (0.46 μg/mL/6 h; Figure 1B) was found to be the most effective, in which cell viability declined to 46.65% in comparison to the control. Beyond that, the cell viability increased to 75% and above. During concentration and time-dependent studies in A549, once again a “U” shaped viability pattern was observed with a different exposure time of BT.

Since mitochondrial membrane potential is important for the maintenance of cellular health and viability, we further checked the effect of BT on mitochondrial homeostasis. The BT-mediated selective cytotoxic effect on the two LUAD cell lines was reconfirmed by the JC-1 staining where a reduced red: green fluorescence intensity was observed to be proportional to the mitochondrial depolarization (Figure 1C).

A wide range of BT (0.046–46.0 μg/ml) treatment for varied periods (12–48 h) did not exert any cytotoxic impact on BEAS-2B cells, but a plateau pattern was observed with a marginal proliferation at lower concentrations (Figure 1D).

### 3.2 BT-mediated chemosensitization towards cisplatin in A549 cells

**FIGURE 2:**
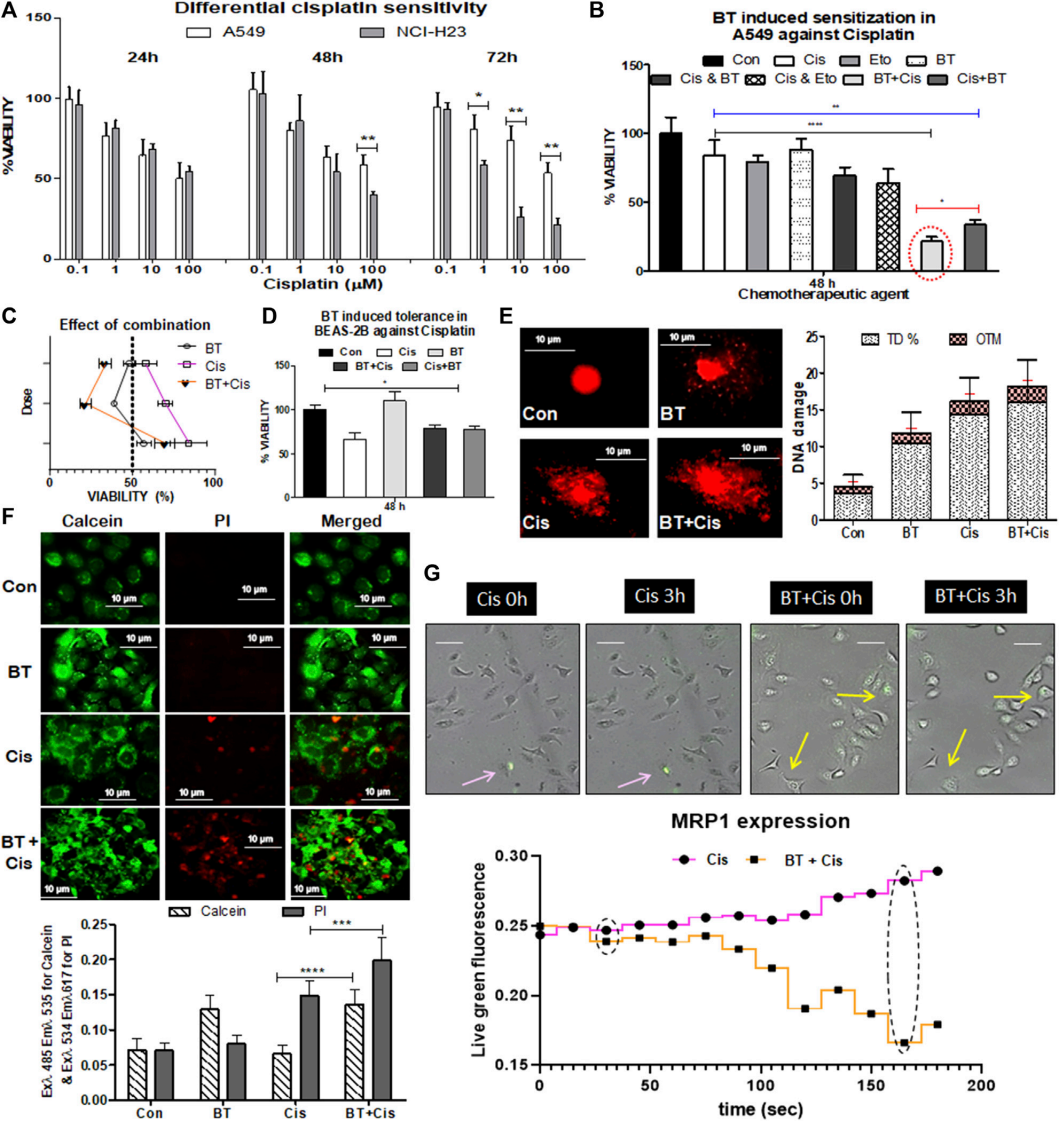
Chemotherapeutic response of LUAD cell lines to cisplatin, and BT-mediated chemosensitization in A549 cells. Concentration- and time-dependent effect of cisplatin on the viability of two lung cancer cell lines NCI-H23 and A549 **(A)**; effect of different small molecular adjuvant (e.g., BT or etoposide) on modulation of cisplatin-toxicity in A549 cells **(B)**; determination of the effect of BT-combined cisplatin on the viability of A549 **(C)**; effect of BT, cisplatin or their different combinations on the viability of normal lung cells BEAS-2B **(D)**; effect of the cytotoxically most potent combination of BT and cisplatin on DNA-damage **(E)**, drug-efflux capacity **(F)** and MRP1 expression in real-time imaging **(G)** of A549 cells. *p < 0.05, **p < 0.01, ***p < 0.001 and ****p < 0.0001, compared with the control. Abbreviations: Con, control; Cis, cisplatin; Eto, etoposide.

Cisplatin (0.1–100 µM), a DNA intra-strand cross-linking agent alone, induced a marked decrease in viability (by 40%–60%) of NCI-H23 cells with a gradual increase in exposure time and concentration (Figure 2A). However, the same drug caused little to moderate toxicity (20%–35%) in A549 cells after 24 and 48 h of treatment with high concentrations. For a higher concentration and a longer period where NCI-H23 showed huge cytotoxicity (IC_50_ 0.85 µM), but A549 remained consistently inert (IC_50_ 8.61 µM) (Figure 2A). Since the IC_50_ value of cisplatin in NCI-H23 cells was 10-fold lower than that of A549, no further study was required for drug concentration minimization and resistance reversal. The resistance of the A549 cells toward cisplatin indicated the requirement of some adjuvants for increasing its toxicity in these cells. A combination of etoposide/BT for drug concentration minimization was observed through co-administration modalities in A549 cells.

The viability of A549 cells with the monotherapy of cisplatin (1.0 µM/48 h) or etoposide (1.0 µM/48 h), or BT (0.46 μg/ml/48 h) was 84%, 79%, and 89%, respectively (Figure 2B). The combinatorial enhancement of cisplatin toxicity by another lung cancer drug, etoposide was 14%, and with that of BT was 22.67% after 48 h concurrent treatment. But surprisingly, a huge 42% and 36.5% reduction in A549 viability was achieved when a short pulse of BT was added either at the beginning or the end of cisplatin treatment (Figure 2B). Shorter exposures (6 h) of BT (0.46 μg/ml) in both sensitization (pre-treatment) and potentiation (post-treatment) modes were significantly effective in increasing the cytotoxic response of A549, proving BT a better and safer adjuvant candidate. The mode of combination could not be determined through the Chou-Talalay equation as the IC_50_ value could not be deduced from this non-linear type of concentration response. But the extent of reduction in viability (<65%) indicated towards synergistic action of the BT with cisplatin pretreatment combination in inducing cytotoxicity which was more than double that of the cisplatin alone (Figure 2C).

When the adverse cytotoxicity of the drug, cisplatin was tested in the normal bronchial epithelial cells BEAS-2B, an interesting finding was obtained. Here the viability was increased from 65.33% to 70%–73% when cisplatin was combined with BT giving more tolerance power in BEAS-2B (Figure 2D). This might have been due to the mild cytoprotective action of the theaflavin-rich BT (0.46 μg/mL/48 h) as seen in Figure 2D. Thus, BT apart from increasing the efficacy of cisplatin in A549 cells exhibited the cytotoxic effect in a cell line-specific manner.

The cisplatin-resistance reversal by BT was tested in resistant A549 cells by assessing DNA-damaging capacity, which is one of the major features of the anticancer efficacy of cisplatin. The DNA damage was studied by alkaline comet assay with high TD% and OTM implying greater damaging ability of the agent. Though BT itself did not induce damage to A549 the BT plus cisplatin pre-treatment enhanced DNA damage, and the improvement was evident (in terms of higher TD% and OTM) in comparison to monotherapy of cisplatin (Figure 2E).

Rapid efflux of the calcein-AM stain by the ABC transporters under the basal condition implied strong multidrug resistance. BT caused enhanced retention of green fluorescence of cleaved calcein stain inside the cells which was indicative of reduced drug efflux, i.e., improved drug efficacy. The rapid drug-efflux nature of cisplatin-treated A549 was significantly reduced when BT was combined with it as seen from the enhanced retention of green fluorescence of cleaved calcein stain along with red fluorescence of death-specific PI stain (Figure 2F). BT introduced a slight alkalinity of extracellular medium where the extracellular pH (pHe), post-cisplatin-treatment was 7.2 ± 0.12 and reached 7.58 ± 0.09 in the case of BT-pre sensitized cisplatin-treatment in A549.

Other than passive diffusion, cellular uptake of cisplatin is facilitated through drug transporters. We have found an early indication of decreased MRP1 expression and cell shrinkage/retraction with the administration of BT plus cisplatin combination in comparison to cisplatin-monotherapy as visualized from time-lapsed live cell imaging cisplatin A549 cells (Figure 2G). This lowering of MRP1, an NRF2-directed drug-efflux pump is likely the probable reason behind this BT-sensitized improved chemo response of cisplatin which needs further validation.

### 3.3 BT-mediated differential modulation of NRF2-KEAP1 machinery in LUAD cells

**FIGURE 3:**
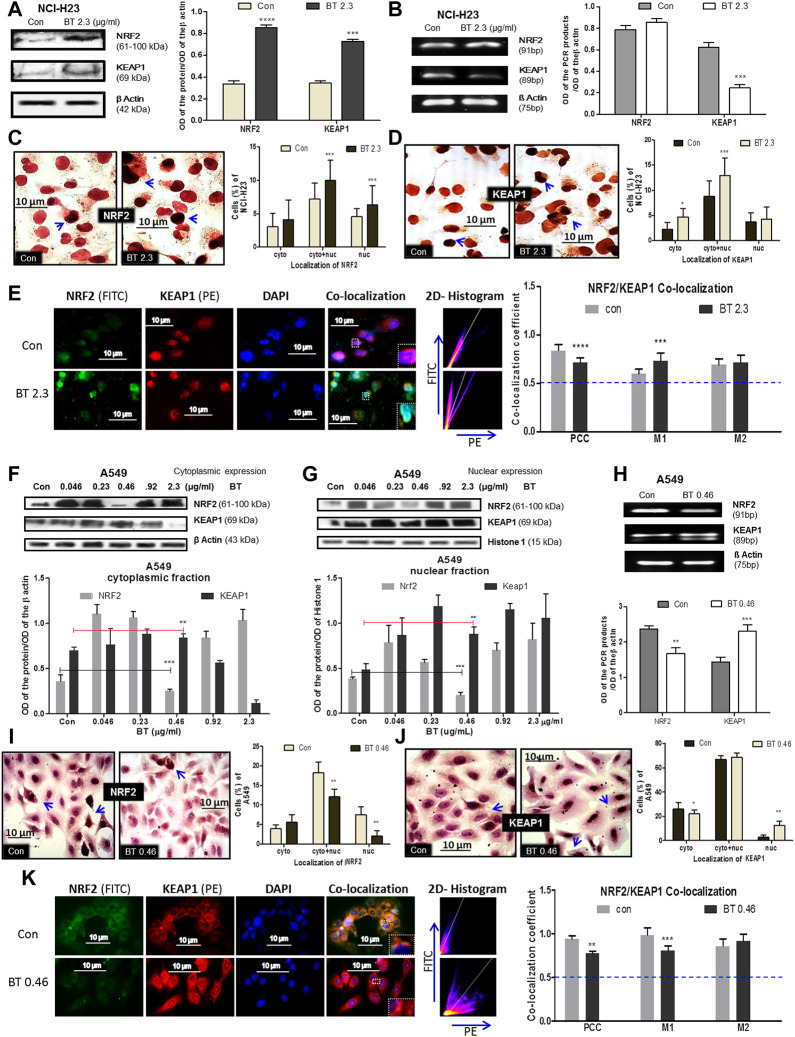
BT mediated differential modulation of NRF2-KEAP1 machinery in NCI-H23 and A549 cells. Effect of BT on NRF2 and KEAP1 protein expression **(A)**; mRNA expression **(B)**; sub-cellular localization of NRF2 **(C)**; KEAP1 **(D)** and their co-localization **(E)** in NCI-H23 cells. Effect of BT on NRF2 and KEAP1 protein expression from cytoplasmic **(F)** and nuclear **(G)** fraction; mRNA expression **(H)**; sub-cellular localization of NRF2 **(I)**; KEAP1 **(J)** and their co-localization **(K)** in A549 cells. *p < 0.05, **p < 0.01, ***p < 0.001 and ****p < 0.0001, compared with the control. Abbreviations: Con, control; BT 0.46, BT 0.46 μg/mL/6 h; BT 2.3, BT 2.3 μg/mL/12 h.

The BT-mediated modulation of the redox regulatory machinery NRF2-KEAP1 was studied in two different KEAP1 mutated LUAD systems NCI-H23 and A549. NCI-H23 with a low basal expression of NRF2 owing to a gain-of-function mutation in KEAP1 responded peculiarly towards BT (2.3 μg/ml/12 h). BT increased NRF2 protein expression (2.83-fold) but could not decrease KEAP1 protein accordingly (Figure 3A), rather a 2.1-fold increase was found. The mRNA expression reflected a similar upregulation of NRF2 by 1.35-fold (Figure 3B) but in contrast to protein, a compensatory downregulation of KEAP1 (2.05-fold) was observed at the transcriptional level (Figure 3B).

From their sub-cellular localization pattern, an interesting observation was made that, BT caused more nuclear accumulation of NRF2 (Figure 3C). On the other hand, the rise in the KEAP1 was more prominent in the cytoplasm (Figure 3D) reconfirming the immunoblot observation. This might have been because excess KEAP1 was trying hard but failed to degrade off the accumulated NRF2.

As proteins NRF2 and KEAP1 physically interact with each other and this interaction very much determines their function, co-localization was studied to measure their interaction. Though the PCC [indication of co-localization of the two interacting proteins (here, NRF2 and KEAP1)] was markedly reduced, it was significant enough to indicate that basal NRF2-KEAP1 interaction was sustained in BT-treated NCI-H23 cells (Figure 3E). The 2D-histogram scattering plot of FITC vs. PE evidenced that the FITC axis had predominance after BT treatment (Figure 3E) which indicated an abundance of NRF2. The distortion in the co-localization pattern was mainly because of BT-induced robust NRF2 expression. The same was also visible with the rise of the MOC 1 (M1) which was assigned for NRF2 (Figure 3E). M2 was not greatly altered.

In A549 the redox modulatory effect of BT on NRF2 and its inhibitor KEAP1 was more or less opposite to that of NCI-H23. The loss of function mutation of KEAP1 rendered constitutive expression of NRF2 which was efficiently controlled by BT in a hormetic manner. BT controlled NRF2 expression in the cytoplasm (Figure 3F) and even more prominently in the nucleus (Figure 3G) again in a U-shaped manner where the concentration of 0.46 μg/mL/6 h showed the highest NRF2 inhibition. This was a reflection of our previous cytotoxicity results. In both the fractions the NRF2:KEAP1 ratio was lowest at this concentration. The stimulatory effect of BT on cytoplasmic and nuclear KEAP1 was consistent.

Similarly, from the mRNA transcripts, we observed that BT (0.46 μg/ml/6 h) significantly downregulated NRF2 by 0.65-fold, and a simultaneous 0.89-fold upregulation in its sensor-cum-adaptor KEAP1 was also evident (Figure 3H). The immunoblot findings were further validated by immunocytochemistry which also showed that at this low concentration, BT reduced the nuclear localization of NRF2 (Figure 3I) and increased that of nuclear KEAP1 (Figure 3J). The nucleus to the cytoplasmic shift of NRF2 and exactly the opposite shift in the case of KEAP1 further supported the hypothesis that BT-induced nuclear export of NRF2 was escorted by KEAP1.

The PCC was once again reduced upon BT treatment but a considerable NRF2-KEAP1 interaction was maintained in A549 (Figure 3K). The distortion in the co-localization pattern was mainly due to the reduced expression of NRF2 protein by BT. The same pattern was reflected with the reduction of the MOC 1 (M1) assigned for NRF2. The M2 was also increased indicating a KEAP1-upregulation but it was not significant. The 2D-histogram scattering plot of FITC vs. PE showed a predominance of PE (Figure 3K) again indicating an absence of NRF2 upon BT exposure.

### 3.4 BT caused alterations of NRF2 transcriptional targets and non-KEAP1 regulators in lung cancer cells

**FIGURE 4:**
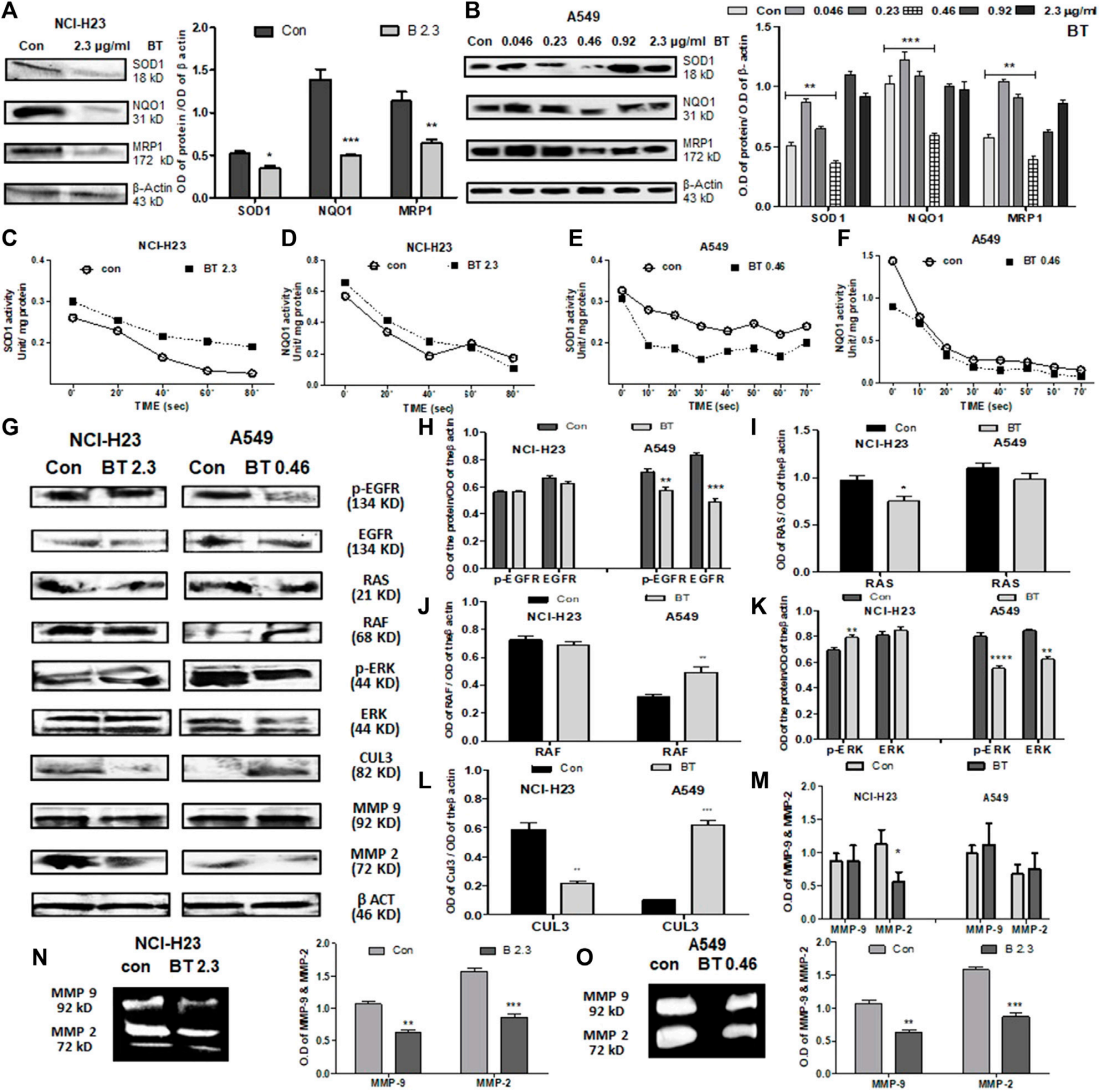
BT caused alterations of NRF2 downstream effectors and upstream regulators in lung cancer cells. Effect of BT on the expression of NRF2 downstream targets-SOD1, NQO1, and MRP1 in NCI-H23 **(A)** and A549 **(B)**; effect of BT on the enzymatic activity of NRF2 downstream molecules SOD1, and NQO1 in NCI-H23 **(C, D)** and A549 **(E, F)**. Effect of BT on the expression of non-KEAP1 NRF2 regulators, phospho-EGFR, EGFR, RAS, RAF, phospho-ERK, ERK, CUL-3, MMP-9, and MMP-2 in NCI-H23 and A549 [**(G–M)**, respectively]; gelatinolytic activity of MMP-9 and MMP-2 in NCI-H23 **(N)** and A549 **(O)**. *p < 0.05, **p < 0.01, ***p < 0.001 and ****p < 0.0001, compared with the control. Abbreviations: Con, control; BT 0.46, BT 0.46 μg/mL/6 h; BT 2.3, BT 2.3 μg/mL/12 h.

In NCI-H23, BT (2.3 μg/ml/12 h) decreased the expression of the downstream molecules of NRF2, namely, SOD1, NQO1, and MRP1. Despite the NRF2 upregulation, NQO1, MRP1, and SOD1 were downregulated at the protein level (Figure 4A) maybe because of the presence of high KEAP1.

BT presented a U-shaped hormetic downregulation of all three NRF2 targets in A549 cells. In congruence with the NRF2-downregulation, the most cytotoxic concentration of BT, i.e., 0.46 μg/mL/6 h, again most efficiently downregulated SOD1, NQO1, and MRP1expression (Figure 4B).

Though BT downregulated the expression of SOD1, its enzymatic activity was significantly enhanced by 1.33-fold in NCI-H23 cells (Figure 4C). This might have been an adaptive response to compensate for the fall in the total SOD1 content in NCI-H23 cells. However, BT caused only a nominal increase in the enzymatic activity of NQO1 in NCI-H23 (Figure 4D). Just like the expressional suppression, BT also inhibited the SOD1 activity by a huge 1.45-fold (Figure 4E) but could not induce the same in the NQO1 activity of the A549 cells (Figure 4F). The unchangeable tendency of robust NQO1 was constant for both A549 and NCI-H23 cells.

When the non-KEAP1 regulators of NRF2 were studied, it was found that BT downregulated the NRF2-activator, EGFR, and phosphorylated -EGFR (Tyr1173) in A549 but its effect was not so evident in NCI-H23 (Figures 4G,H). RAS and its downstream RAF, two NRF2-activatory kinases that facilitate the NRF2 acted differently in NCI-H23 and A549. RAS remained almost unchanged (Figures 4G,I) whereas RAF was highly activated (Figures 4G,J) by BT treatment in A549. On the other hand BT downregulated RAS (Figures 4G,I) but could not show any alteration in RAF (Figures 4G,J) in NCI-H23. ERK, the RAF-downstream, and another NRF2 activator was also suppressed by BT in A549 (Figures 4G,K), and the effect was more prominent in the functionally active phosphorylated forms (Thr 202, Tyr 204) or p-ERK. In NCI-H23, BT influenced the rise in p-ERK (Thr 202, Tyr 204) (Figures 4G,K).

BT showed a distinct regulatory effect on the KEAP1 upstream NRF2 ubiquitination complex Cul-3 (Figures 4G,L) in A549 and NCI-H23. In A549 the upregulation in Cul3 converged with the NRF2-inhibition. However, the strange rise of the KEAP1 in NCI-H23 got an explanation for the downregulation of Cul3 which is the counterpart of the NRF2 degradation/ubiquitination complex. Cul3 got markedly diminished with BT treatment in NCI-H23, and therefore, KEAP1 might have triggered a subsequent adaptation to cope with elevated NRF2.

The BT-mediated expressional downregulation in MMP-2 at the protein level was evidenced only in NCI-H23, and not in A549 (Figures 4G,M). MMP-9 expression was not altered with BT treatment either in NCI-H23 or in A549 cells. However, BT was effective in suppressing MMP-2 and MMP-9 activity both in A549 (Figure 4N) and NCI-H23 cells (Figure 4O). However, the inhibitory effect was more prominent in NCI-H23 which might have been due to the relatively higher concentration of BT in NCI-H23 cells.

### 3.5 Comparison of the effect of synthetic NRF2-modulators and BT on NRF2/KEAP1 and their upstream regulators in NCI-H23 and A549 cells

**FIGURE 5:**
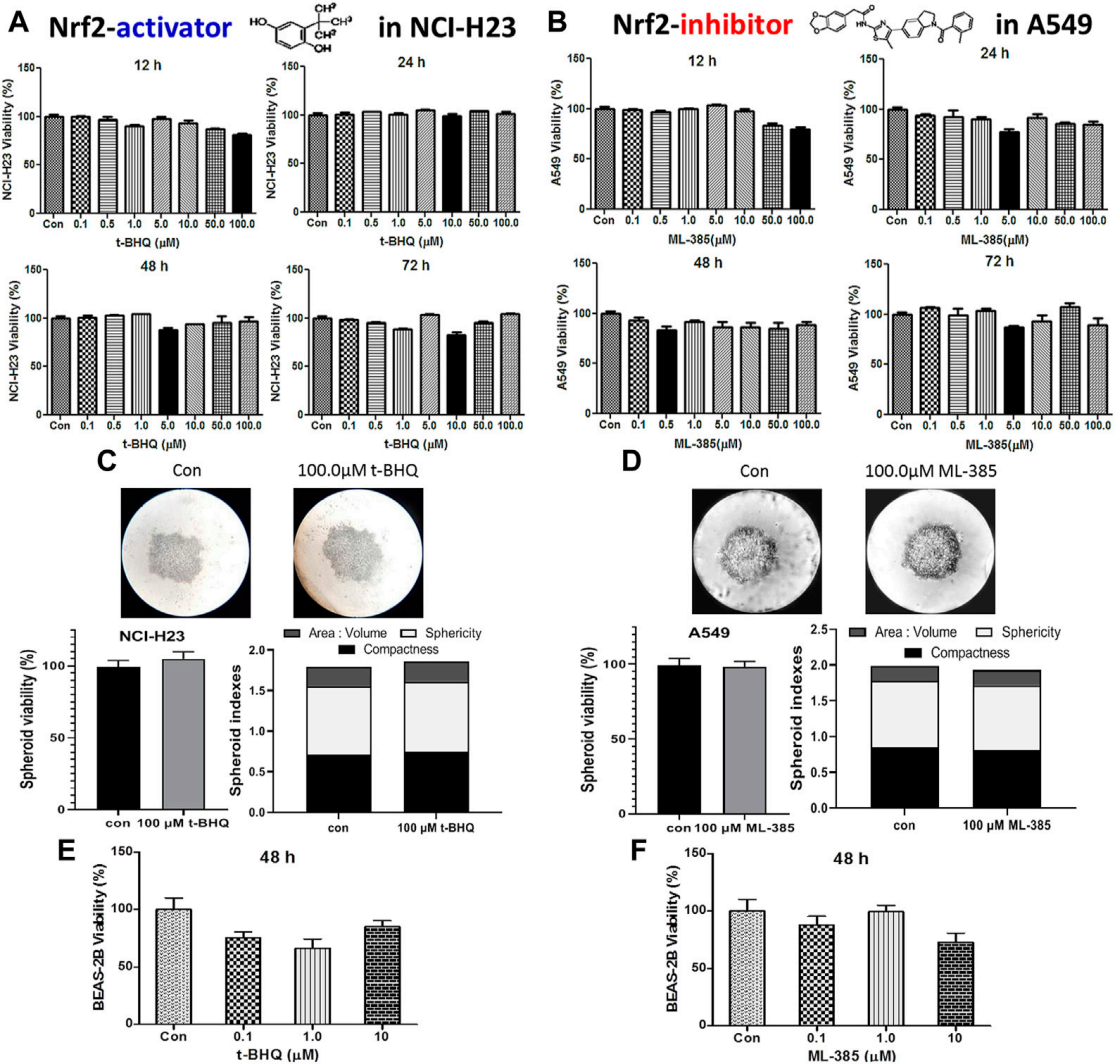
Cytotoxic efficacy of t-BHQ and ML-385 in different lung cells. Effect of t-BHQ and ML-385 on the viability of two lung cancer cell lines NCI-H23 **(A)** and A549 **(B)** cells, respectively, as 2D monolayer; effect of t-BHQ and ML-385 on viability and spheroid indexes such as area: volume, sphericity and compactness of the 3D spheroid of NCI-H23 **(C)** and A549 **(D)** cells, respectively; Differential sensitivity of normal bronchial BEAS-2B cells against t-BHQ **(E)** and ML-385 **(F)**. *p < 0.05, **p < 0.01, ***p < 0.001 and ****p < 0.0001, compared with the control. Abbreviations: Con, control.

The cytotoxic effect of the NRF2 activator, t-BHQ was investigated in NCI-H23 cells. The activator did not show any significant cytotoxicity with the varied concentration and time in monolayer (Figure 5A) and spheroid culture (Figure 5C). The cytotoxic effect of NRF2 inhibitor, ML-385 was investigated in A549 cells. Here again, the agent did not show any cytotoxicity with the variation of concentration and time in monolayer (Figure 5B) and spheroid culture (Figure 5D). Even in the BEAS-2B cells, the NRF2-modulators did not elicit any significant toxicity (Figures 5E,F).

**FIGURE 6:**
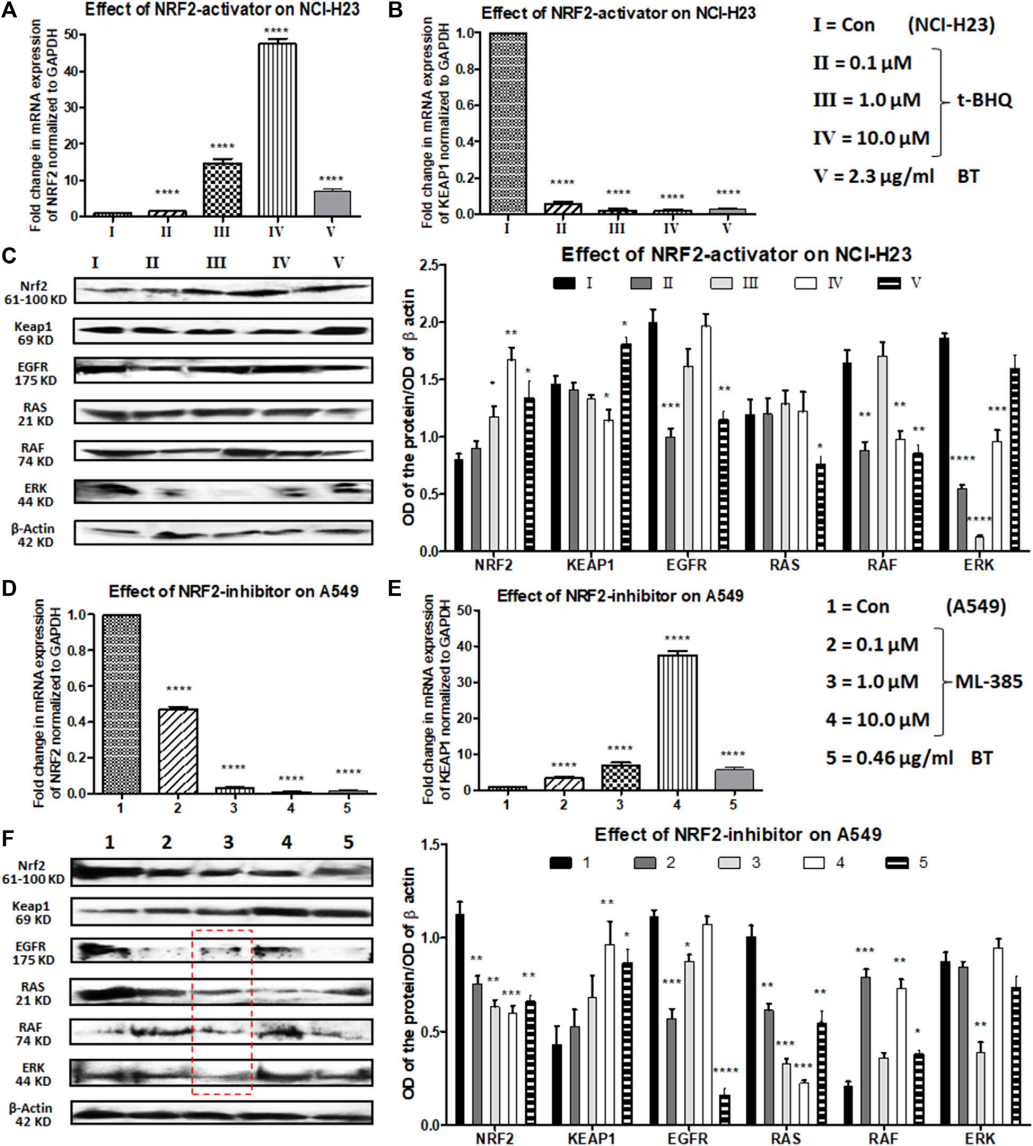
Comparison of the effect of t-BHQ/ML-385 and BT on NRF2/KEAP1 and their upstream regulators in NCI-H23 and A549 cells. Effect of t-BHQ and BT on mRNA expression of NRF2 **(A)** and KEAP1 **(B)**; protein expression of NRF2/KEAP1 and their regulators EGFR-RAS-RAF-ERK **(C)** in NCI-H23. Effect of ML-385 and BT on mRNA expression of NRF2 **(D)** and KEAP1 **(E)**; protein expression of NRF2/KEAP1 and their regulators EGFR-RAS-RAF-ERK **(F)** in A549. *p < 0.05, **p < 0.01, ***p < 0.001 and ****p < 0.0001, compared with the control. Abbreviations: Con: control.

In NCI-H23 (normally NRF2 is suppressed), a concentration-dependent NRF2-upregulation and a simultaneous KEAP1 downregulation were achieved with a pharmacological NRF2-activator, t-BHQ at transcriptional as well as translational levels (0.1–10 μM). The concentration-dependent NRF2 upregulation and KEAP1 downregulation were more prominent at the mRNA levels (>10-fold change) (Figures 6A,B) than at the protein expression levels (Figures 6C,D). BT showed similar efficacy as t-BHQ in increasing NRF2, but not in decreasing KEAP1 protein level in NCI-H23 cells. Although the NRF2-activation by BT (2.3 μg/mL/12 h) was not as good as the highest concentration of synthetic NRF2-activator (10 μM/24 h) it caused significant alterations at both mRNA (Figures 6A,B) and protein levels (Figure 6C).

EGFR, RAS, RAF, and ERK apart from being oncogenic drivers, positively regulate the expression and activity of NRF2. After a slight initial decline with a 0.1 μM concentration of t-BHQ, a gradual rise in EGFR expression was observed with increasing concentrations of t-BHQ in NCI-H23 cells (Figure 6C). The EGFR downstream and another NRF2-inducer RAS remained more or less unaltered with t-BHQ in NCI-H23 cells (Figure 6C). However, BT (2.3 μg/mL/12 h)-mediated significant reduction in EGFR and its downstream signaling RAS (Figure 6C) in NCI-H23 cells was satisfactory. RAF expression was gradually upregulated with t-BHQ (10.0 µM) and ERK was downregulated by t-BHQ (1.0 µM) in NCI-H23 cells (Figure 6C). On the contrary BT (2.3 μg/mL/12 h) depleted RAF more prominently than ERK in NCI-H23 cells which were indicative of improved anticancer outcomes in NCI-H23 cells.

ML-385 concentration-dependently reduced expression of NRF2 and simultaneously induced expression of its inhibitor KEAP1 in NRF2-upregulated A549 cells. In terms of relative fold change (2^^−ΔΔCt^), a huge reduction in NRF2 mRNA level and a parallel induction in KEAP1 mRNA level were evidenced with quantitative real-time PCR (Figures 6D,E). The highest concentration of NRF2 inhibitor, ML-385 (10.0 μM) caused a significant downregulation of NRF2 and a similar upregulation of KEAP1 protein expression. BT and ML-385 were equally robust in inhibiting the mRNA expression of NRF2 (again >10-fold change). A simultaneous KEAP1 activation was also seen with BT (0.46 μg/ml/6 h) treatment in NRF2-hyperactivated A549 (Figure 6E). The translational modulation of both NRF2 and KEAP1 by BT was exactly similar to their transcriptional alteration (Figures 6D–F).

There were interesting modulations in NRF2-regulatory machinery by both types of inhibitors, ML-385 (0.1–10 μM) and BT in A549. There was an overall reduction in the EGFR expression with ML-385 treatment, but the lowest concentration, i.e., 0.1 μM showed the best minimization effect (Figure 6F). An intermediate concentration of ML-385 (1.0 μM) proved overall most effective in the inhibition of all the NRF2 activators, namely, EGFR, RAS, RAF, and ERK, in A549 cells (Figure 6F). Apart from ERK, BT (0.46 μg/mL/6 h) was equally effective as ML-385 in A549 to block the NRF2 activation pathway of EGFR/RAS/RAF (Figure 6F) proving its multimodal antineoplastic activity.

## 4 Discussion

The NRF2/KEAP1 signaling which is truly defined as the “guardian of healthspan” (Lewis et al., 2010) is quintessential for the healthy maintenance of normal eukaryotic cells as it regulates the transcription of >500 cytoprotective genes containing antioxidant responsive element (ARE) sequence (Bellezza et al., 2018). This NRF2-KEAP1-ARE system gives direct anticarcinogenic protection against cellular oxidative and xenobiotic stress (Wu et al., 2019). But the deregulation of the same NRF2-KEAP1-ARE system provides stress defense to the aggressive cancerous cells against all sorts of anticancer therapies, making them much more unmanageable. Therefore, reorientation of this dual nature of NRF2 has become a keystone for therapeutic success.

The deadliness of lung cancer lies in the wide spectrum of resistance against routinely used therapies (Roche, 2021). Though the proportion of TR is higher than that of TF in the commonly used water-extract of black tea, several *in vitro* and *in vivo* studies have shown that the TF fraction of BT owing to its abundant hydroxyl groups is chiefly responsible for inducing apoptosis, growth arrest, DNA damage, and other prominent chemotherapeutic effects in lung cancer models (Imran et al., 2019). With this background, we had chosen the black tea extract with an excess of TF (>80%), as the investigational compound against LUAD. Most of the available BT-mitigated *in vitro* NSCLC studies (Wang et al., 2011) have used supraphysiological high concentration (e.g., 100 μM) for a prolonged period (≥24 h) (O’Neill et al., 2021), but in reality, due to its poor bioavailability and rapid biotransformation, the half-life of most tea polyphenols remain 6–12 h (Chow and Hakim, 2011) and their peak plasma concentrations are in low micromolar ranges (Pereira-Caro et al., 2017). In this context, we have shown the implication of transient exposure to a physiologically relevant concentration of BT in different lung cells where the NRF2 activity is deregulated because of different KEAP1 mutations. In an intrinsically non-responsive state, the quick introduction of many natural compounds including tea flavonoids in their submicromolar concentrations have remarkably improved toxicity. Such pulsatile augmentation incurs a unique J-/U-shaped stress response instead of the linear concentration-response which is called hormesis (Gezer, 2018). In this work, BT has shown this biphasic behavior in viability and subsequent stress modulation of the NRF2-deregulated LUAD cells.

Despite the side effects of chemotherapy, cisplatin-based high-dose monotherapy or its combination with etoposide is still the backbone of locally advanced NSCLC (including LUAD) treatment. But after an initial success in more than 20% of cases, mutation-governed resistance and/or recurrence becomes the major challenge to its therapeutic success (Li et al., 2020). A549, a LUAD cell line with G333C loss of function mutation of KEAP1, was resistant, while NCI-H23, another LUAD cell with Q193H gain of function mutation of KEAP1 (Probst et al., 2015) was responsive to the chemotherapeutic drugs, such as etoposide and cisplatin. BEAS-2B with wild-type KEAP1 showed intermediate resistance towards cisplatin. KEAP1 mutations render therapy and/or stress resistance in lung cancer and the above-mentioned differential cisplatin response was a direct reflection of their NRF2-KEAP1 status (Kansanen et al., 2013). BT, a multifunctional anticancer phytomixture can induce oxidative stress selectively in cancer cells resulting in stress-mediated apoptosis *via* mitochondrial depolarization, thiol modification of cysteine residues, and enhanced killing. Increased modification of the cellular thiols is a mode of cisplatin resistance, especially at lower doses (Sadowitz et al., 2002). BT was reported to antagonize cisplatin resistance by lowering the thiol-rich KEAP1 (Torigoe et al., 2005). In congruence with this, we observed that KEAP1, a cysteine-rich molecule was modulated by BT in such a way that mitochondrial depolarization was achieved and more vividly in high KEAP1-expressing NCI-H23. But the BT-mediated cytotoxicity was most prominent in cancerous A549, moderate in NCI-H23, and least effective in normal BEAS-2B cells.

BT or its components can synergistically improve the therapeutic efficacy of chemotherapeutic drugs when used as an adjuvant in cancer, especially in LUAD cells (Roche, 2021; Datta et al., 2022). Since cisplatin-induced significant cytotoxicity in NCI-23 cells at low doses, further drug sensitization studies were not taken up. Neither etoposide (another common NSCLC drug) nor BT co-administration was potent enough in inducing cytotoxicity in A549 cells. There was no substantial increase in the cisplatin-toxicity in A549 when BT/etoposide was combined with the cisplatin for a long 48 h of exposure. Our earlier work reported etoposide resistance may be minimized by the principal green tea constituent, epigallocatechin gallate (EGCG) (Datta and Sinha, 2019). But very interestingly a short 6 h exposure of BT (0.46 μg/mL) in A549 immediately before the cisplatin treatment markedly enhanced its sensitivity as evidenced by the increased DNA damage and diminished drug-efflux ability of BT-sensitized cisplatin. It is possible that the early trend of time-dependent decrease in MRP1, an NRF2-directed drug-efflux pump, was the probable reason behind this BT-sensitized improved chemoresponse of cisplatin which needs further validation. When we studied the pH_e_ of BT-pretreated and untreated cisplatin-exposed cells, a clear rise in pH_e_ was observed which might have been due to the pro-oxidative nature of BT. The basic pH of the extracellular medium might have facilitated the activity of the alkaline chemo drug, cisplatin, against the A549 cells. Moreover, it has been reported that at lower intracellular pH, cisplatin acts better (Shirmanova et al., 2017)_._ Interestingly, the BT-pretreated normal BEAS-2B cells tolerated the adverse toxicity of the drug efficiently.

Since cisplatin-response was directly linked with NRF2-KEAP1 status in the two LUAD cell lines, the next step was a detailed mechanistic study of the modulation of NRF2/KEAP1 machinery with BT. As the KEAP1 mutation in A549 was in the Kelch-I repeat (327–372) domain and that of NCI-H23 was in the intervening (IVR) region (180–315), there was room for KEAP1-NRF2 interaction in A549 and KEAP1-CUL3 interaction in NCI-H23, respectively (Canning et al., 2015). Therefore, the scope of tea-mediated case-specific NRF2-modulation remained unaffected by those mutations in these 2 cell lines. However, BT-influenced divergent regulation of NRF2 in NCI-H23 and A549 cells. Though BT-induced downregulation of NRF2 was concorded with the upregulation of KEAP1 in A549, there was an ambiguous upregulation of KEAP1 even in the presence of robust NRF2 activation in NCI-H23. According to the KEAP1-CUL-3 dissociation model, the binding of inhibitory complex KEAP1 and CUL-3 is disrupted due to electrophile-induced cysteine thiol modifications in a redox-specific manner, leading to the escape of NRF2 from the ubiquitination system (Canning et al., 2015). In consilience with this, the downregulation in ubiquitin-mediated NRF2-degrading machinery, CUL-3, might have necessitated a compensatory stabilization of its ubiquitination-counterpart KEAP1 despite the strong upregulation of NRF2.

Apart from NRF2, its plethora of downstream targets play an important role in augmenting drug resistance in cancer. Therefore, the selection of NRF2 targets was based on their contribution to resistance development. MRP1 is primarily involved in drug efflux in various cancer cells (Lu et al., 2015) whereas NQO1 and SOD1 upregulation increases drug resistance making the prognosis even worse (Datta and Sinha, 2019). However, NRF2-upregulation was not translated into the expression of downstream targets, such as SOD1, NQO1, and MRP1, by BT in NCI-H23. But the rise in the catalytic activity of SOD1 and NQO1 may have counterbalanced their expressional repression. In A549 the response modulatory effect of BT on the redox-regulator NRF2 and its prime inhibitor KEAP1 was comparable to our previous work with green tea polyphenol, EGCG (Datta and Sinha, 2019). The constitutive expression of NRF2 in A549 was efficiently tackled by brief exposure to BT where the maximum NRF2-suppression and KEAP1-promotion were observed with the cytotoxically most potent concentration of BT (0.46 μg/ml/6 h). In the case of A549, the depleted expression of NRF2 caused an expected reduction in its downstream molecules both in terms of expression and activity. BT-mediated regulation was not very effective in modulating the NQO1 activity in either of the cell lines. NQO1 is one of the most robustly upregulated molecules in NSCLC cells than benign or even other types of lung cancer cells (Liu et al., 2012).

EGFR regulation, the most-reported target of BT, plays a pivotal role in the NRF2 signaling cascade. BT catechins, especially TF have been reported to effectively alter EGFR and downstream target ERK to exert chemotherapeutic impact in different cell cultures and animal models (Pan et al., 2013). In our study, the TF-rich BT caused a modulation of NRF2-activatory kinase-ERK which was found to be in the opposite direction in the two LUAD cells, i.e., it was enhanced in NCI-H23 and attenuated in A549. It can be explained by the fact that activation of ERK resulted in NRF2 stabilization and rapid nuclear translocalization (Panieri and Saso, 2019). To our surprise, the ERK modulation was not parallel with that of RAF in either of the cell lines. The reason might have been a mutation in its immediate upstream RAS (Yoon et al., 2010) which might have rendered RAF unresponsive in both scenarios. The effect of non-canonical NRF2 inducers, (EGFR and ERK) was much more prominent in A549 than in NCI-H23.

In the present study, we aimed to elucidate the impact of BT on MMP activity in NCI-H23 and A549, to strengthen the anticancer outcome. BT was potent enough to decrease the activity and expression of these gelatinolytic MMPs (MMP-2 and MMP-9) but the impact was much greater in NCI-H23. BT catechins have been found to prevent metastasis by downregulating EGFR/ERK signaling-mediated MMP expression and activity in highly metastatic melanoma models (Sil et al., 2010). p-ERK plays the main role in MMP regulation, especially in the MMP-9 activation (Arai et al., 2003). In our work also the MMP suppression may be correlated with EGFR downregulation. (Zagorski et al., 2013; Singh et al., 2016).

ML-385 and t-BHQ are the two synthetic NRF2-modulators with known *in vitro* suppressing and inducing effects respectively. In contrast to BT, neither of the two above-mentioned agents had any cytotoxic effects in A549 and NCI-H23 cells. Their non-toxic concentrations were efficient enough to manipulate NRF2 (downregulated in A549 and upregulated in NCI-H23) in a concentration-dependent way. As per reports, both modulators have a direct effect (activation by t-BHQ and inhibition by ML-385) on NRF2-downstream targets in cancer cells (Kansanen et al., 2013; Probst et al., 2015). Therefore, we were more interested in investigating the upstream regulators. The canonical (KEAP1) and non-canonical (EGFR, RAS, RAF, and ERK) regulators of NRF2 were also uniquely modulated with these pharmacological interventions. In comparison to the synthetic modulators of NRF2, BT was observed to be equally potent or even better than t-BHQ/ML-385 in the overall reorientation of NRF2 and its regulators as well as causing sufficient cytotoxicity in lung cancer cells.

It may be summarized that the dual nature of BT was able to reorient the NRF2 axis according to the specific KEAP1 mutation harbored by the two different LUAD cell lines, NCI-H23 and A549. In NCI-H23 where NRF2 was suppressed due to the gain of function of KEAP1, BT aggravated the NRF2 expression, and in A549 where NRF2 was constitutively upregulated due to the loss of function of KEAP1, the same BT attenuated NRF2 expression. In response to BT, the KEAP1-mediated NRF2-reorientation was probably nuclear export in A549 and cytoplasmic sequestration in NCI-H23. Apart from cell-specific KEAP1 mutation, the bidirectional modulation of the NRF2 axis by BT was also dependent on its exposure pattern. The transient exposure to the physiologically relevant concentration of BT made the resistant A549 cells susceptible to cisplatin toxicity. BT counteracted drug resistance by promoting enhanced drug retention, DNA damage, and reduced drug efflux properties. BT regulated both KEAP1-dependent and KEAP1-independent (EGFR/RAS/RAF/ERK) regulation of NRF2 which were further validated with synthetic modulators of NRF2. However, the efficacy of BT in counteraction of drug resistance in LUAD needs further investigations *in vivo* models.

## 5 Conclusion

**FIGURE 7:**
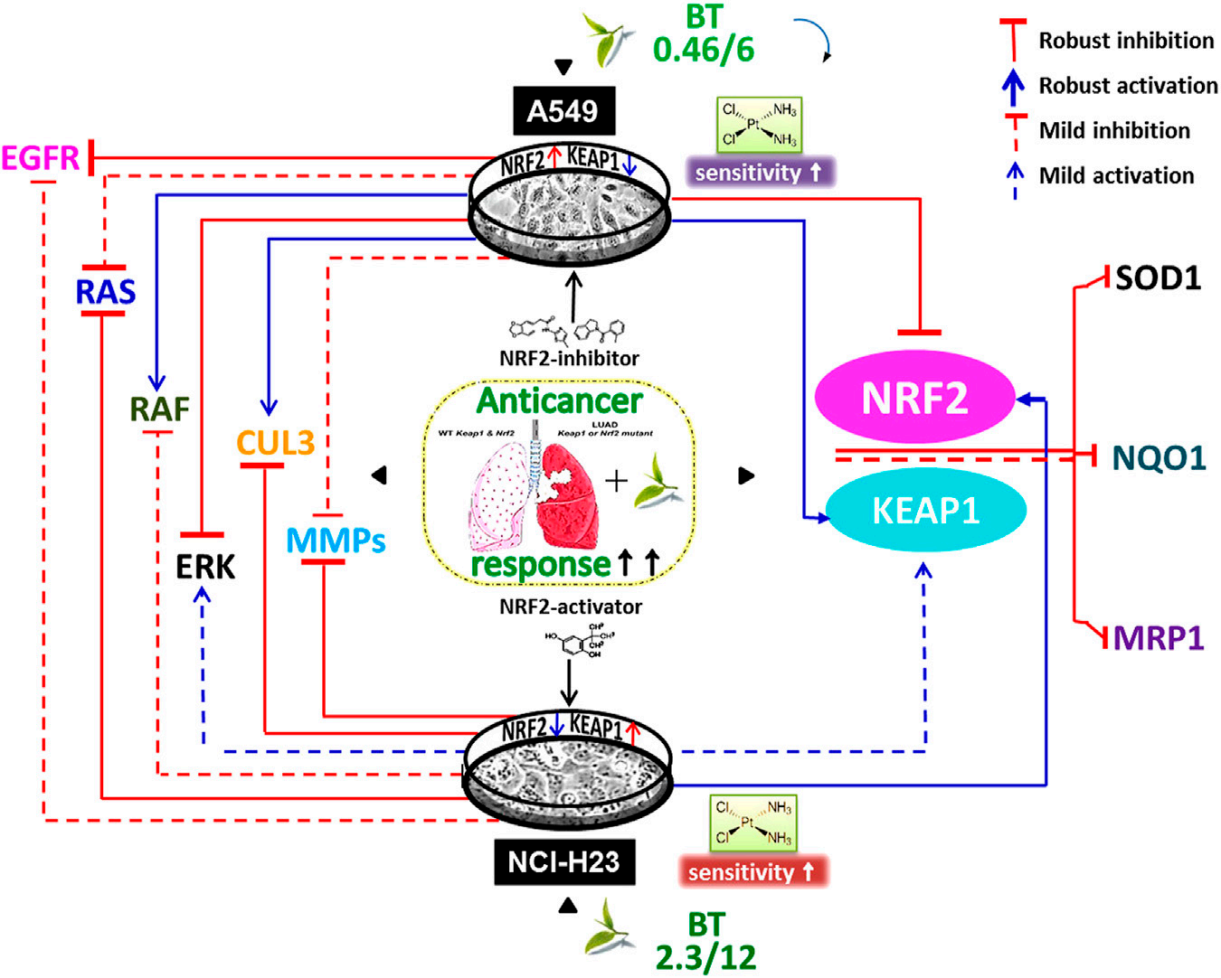
Black tea mediated enhancement of anticancer response via NRF2- reorientation in LUAD scenario. Different exposure of the same black tea components (BT) has realigned the master stress-responsive transcription factor Nrf2 as well as its upstream regulators (e.g., EGFR, RAS, RAF, ERK, MMP-2, and MMP-9) and downstream effectors (e.g., SOD1, NQO1, and MRP1) in two differently KEAP1-mutated LUAD cell-lines (e.g., A549 and NCI-H23). Irrespective of the nature of the NRF2-modulation, there was an overall improved anticancer response in those two opposite LUAD scenarios where the basal drug (e.g., cisplatin) response was a reflection of their NRF2/KEAP1 status and BT has further synergized the cisplatin sensitivity in resistant cells, i.e., A549.

In this study, we have shown for the first time that in the absence of wild-type KEAP1, the NRF2-deregulated LUAD cells have uniquely responded to the dual-nature BT treatment so that NRF2-KEAP1 homeostasis was regained (Figure 7). When the unique composition of individual anticancer polyphenols was briefly used in physiologically low concentration, their cumulative anticancer effect was evident in two oppositely oriented LUAD cells. Better therapeutic outcomes in terms of enhanced drug retention, DNA damage, and reduced MDR-like property and invasiveness were evidences of BT-synergised chemotoxicity. This cisplatin sensitivity (Figure 7) was highly cancer-specific and further dependent on the basal NRF2-KEAP1 status. The NRF2-mediated chemoresponse is under the direct control of its transcriptional descendants (MRP1, NQO1, and SOD1). All these resistance-promoting Nrf2-downstream targets were dampened with BT (Figure 7). But the lowering effect was much more prominent in A549 than NCI-H23 where basal NRF2 was already very high. NRF2-regulatory networks (EGFR, RAS, RAF and ERK) which are also the driving factors of the adenocarcinomic process were also efficiently tackled by TF-rich BT, but the mode of alignment was more or less opposite in the two LUAD cell lines depending on the dual anti-versus pro-oxidant nature of BT phytochemicals (Figure 7). This is the first in-depth report of BT-mediated NRF2 realignment in different LUAD cells with detailed mechanistic underpinning where achievement of an improved yet personalized anticancer effect was the ultimate aim. Therefore, BT may be indicated as a cancer-specific multi-modal small molecule for increasing drug responsiveness in LUAD cells by maintaining NRF2/KEAP1 signaling at an optimum threshold level (Figure 7). It is expected that BT as a potent and selective NRF2-modulator can be further translated into a promising anticancer agent either as a single agent or in combination with other cancer chemotherapeutics.

## Data Availability

The original contributions presented in the study are included in the article/supplementary files, further inquiries can be directed to the corresponding authors.
